# Adaptive enhancement of learning protocol in hippocampal cultured networks grown on multielectrode arrays

**DOI:** 10.3389/fncir.2013.00087

**Published:** 2013-05-24

**Authors:** Alexey Pimashkin, Arseniy Gladkov, Irina Mukhina, Victor Kazantsev

**Affiliations:** ^1^Department of Neurodynamics and Neurobiology, Lobachevsky State University of Nizhny NovgorodNizhny Novgorod, Russia; ^2^Normal Physiology Department, Nizhny Novgorod State Medical AcademyNizhny Novgorod, Russia; ^3^Laboratory of Nonlinear Processes in Living Systems, Institute of Applied Physics of the Russian Academy of ScienceNizhny Novgorod, Russia

**Keywords:** multielectrode arrays, hippocampal cultures, closed-loop, learning *in vitro*, learning in neural networks

## Abstract

Learning in neuronal networks can be investigated using dissociated cultures on multielectrode arrays supplied with appropriate closed-loop stimulation. It was shown in previous studies that weakly respondent neurons on the electrodes can be trained to increase their evoked spiking rate within a predefined time window after the stimulus. Such neurons can be associated with weak synaptic connections in nearby culture network. The stimulation leads to the increase in the connectivity and in the response. However, it was not possible to perform the learning protocol for the neurons on electrodes with relatively strong synaptic inputs and responding at higher rates. We proposed an adaptive closed-loop stimulation protocol capable to achieve learning even for the highly respondent electrodes. It means that the culture network can reorganize appropriately its synaptic connectivity to generate a desired response. We introduced an adaptive reinforcement condition accounting for the response variability in control stimulation. It significantly enhanced the learning protocol to a large number of responding electrodes independently on its base response level. We also found that learning effect preserved after 4–6 h after training.

## INTRODUCTION

Neuronal networks formed in dissociated cultures grown on multielectrode arrays have been widely used as a biological model to monitor mechanisms of information encoding, synaptic plasticity, memory formation, and learning at the network level *in vitro* (le Feber et al., 2010; Frega et al., 2012; Maccione et al., 2012). Planar microelectrode systems permit simultaneous recording and electrical stimulation in different parts of the cultured neuronal network (Thomas et al., 1972).

After 2–3 weeks of spontaneous development the cultured neural networks display spontaneous burst discharges. The discharges consist of 0.1–1 Hz sequences of population bursts of 50–300 ms duration. Recent investigations showed that spatio-temporal patterns of spiking activity within the bursts are organized in a statistically repeatable and reproducible way (Raichman and Ben-Jacob, 2008; Pimashkin et al., 2011). Such repeatability indicated the presence of quite stable synaptic connectivity formed in the cultured network. External electrical stimulation modified the spiking pattern and, hence, induced long-term changes in the synaptic architecture of the underlying network. If the stimulation is applied with closed-loop conditions such changes may be directed to achieve a predefined profile of the evoked response. The latter can further be associated with navigating robots capable to implement simple behavioral tasks (Chao et al., 2008; Shahaf et al., 2008).

Low-frequency electrical stimulation in the form of pulse train (0.03–0.1 Hz) induced population burst responses over most of the neurons in the network during 50–300 ms after the stimulus artifact (Maeda et al., 1995; Wagenaar et al., 2004). Such stimulation did not change functional characteristics of the evoked response at both short and long-term periods (Chiappalone et al., 2008). However, spontaneous bursts can change their pattern after the low-frequency stimulation indicating changes in the network connectivity (Brewer et al., 2009; Bologna et al., 2010; Ide et al., 2010; le Feber et al., 2010). Increasing the stimulation frequency up to 1 Hz or higher led to suppression of the evoked responses (Jimbo et al., 1993; Shahaf and Marom, 2001; Eytan et al., 2003; Wagenaar et al., 2005; le Feber et al., 2010). Note, that tetanic stimulation with 10 Hz induced spike timing-dependent plasticity (STDP) in the culture network (Wagenaar et al., 2006a,b). Note also, that if signal propagation through synaptic pathways was blocked by applying 6-cyano-7-nitroquinoxaline-2,3-dione (CNQX) and (2R)-amino-5-phosphonovaleric acid (APV), the antagonists of *N*-methyl D-aspartate (NMDA) and α-amino-3-hydroxy-5-methyl-4-isoxazolepropionic acid (AMPA)-receptors, then the evoked spikes can be observed only at latencies shorter than 10 ms (Wagenaar et al., 2004). They represent a direct response on the excitation of an axon passing both the stimulation and the recording electrode, or on the excitation of a cell whose axon passes the recording electrode. Blocking Na^+^ channels by tetrodotoxin (TTX) abolished all spontaneous and evoked activity in culture network. These results suggested that in normal conditions the stimulus evoked spikes with the latencies greater than 10 ms represented “network” spikes generated by signal propagation through the synaptic pathways of the culture network.

A closed-loop protocol of learning in cultured network of cortical neurons stimulated by low-frequency signal (0.3–1 Hz) was proposed by Shahaf and Marom (2001). Each stimulus response was defined as a number of evoked spikes appeared in 50 ± 10 ms post-stimulus interval. For continuous stimulation they introduced the response-to-stimulus ratio (R/S) for the single electrode. This quantity was defined as a moving average over 10 preceding responses. It characterized slow changes in the response caused by plasticity of synaptic pathways between neurons located near stimulating and recording electrodes. If the R/S value exceeded a certain threshold (R/S = 0.2 in Shahaf and Marom, 2001) the stimulation was stopped for 5 min providing the reinforcement. Then the cycle was repeated several times. Time interval needed to reach the threshold in each cycle was treated as adaptation time. The decrease of the adaptation time during the stimulation cycles was then interpreted as learning. Contrariwise, low-frequency stimulation in conditions without the reinforcement (e.g., open-loop conditions) did not induce the learning effect. Changes in the response was observed only on the trained electrode, whereas such effect was not found on the other electrodes. le Feber et al. (2010) found that closed-loop stimulation in cortical cultures induced significant changes in synaptic connectivity in contrast to the open-loop conditions. It was also noted that after training the spontaneous bursts were changed enhancing their correlation and synchrony (Li et al., 2007). This learning protocol was used in several other studies (Marom and Shahaf, 2002; Stegenga et al., 2009). It is important to note that only low-active electrodes recording one spike per 10 stimuli (e.g., with R/S = 0.1) were used for learning. Long-term changes were monitored for more than 30 cycles of stimulation. Electrodes with higher R/S (R/S = 0.5) were also examined for learning, but the learning effect was observed only during first six cycles of stimulation (Staveren et al., 2005).

In this paper we presented our results of learning experiments in hippocampal cultured networks on multielectrode arrays with closed-loop stimulation. Using adaptive and activity dependent reinforcement condition we found that the electrodes with relatively high response activity (R/S > 0.1) can be used for learning. Thus, the closed-loop stimulation could modify culture network synaptic pathways with relatively strong connections typically formed in spontaneous development. We also showed that the adaptive reinforcement significantly enhances the number of highly respondent electrodes (typically more than 50%) relative to the ones with lower response (R/S < 0.1) used in the previous studies.

## MATERIALS AND METHODS

### CELL CULTURING

Cell cultures were prepared from the hippocampus of C57BI6 mice embryos at 18th prenatal day (E18) following standard procedures (Potter and DeMarse, 2001; Pimashkin et al., 2011). After trypsin treatment cells were dissociated by trituration and plated on 64-electrode arrays (Alpha MED Science, Japan), pre-coated with adhesion promoting molecules of polyethyleneimine (PEI). The final density of cell culture was about 15,000–20,000 cells/mm2. Note that in previous studies researchers used cultures with cell density of about 10,000–50,000 cells/mm2 (Shahaf and Marom, 2001) and 5000 cells/mm2 (le Feber et al., 2010). In both studies the cultures were plated from cortical cells. In similar learning experiments with hippocampal cultures the density was 2000 cells/mm2 (Li et al., 2007).

Cells were stored in culture neurobasal medium (Invitrogen 21103-049) with B27 (Invitrogen 17504-044), Glutamine (Invitrogen 25030-024) and fetal calf serum (PanEco κ055), under constant conditions of 37°C, 100% humidity, and 5% CO2 in air in an incubator (MCO-18AIC, SANYO). No antibiotics or antimycotics were used. Glial growth was not suppressed because glial cells were essential to long-term culture health. One half of the medium was changed every 2 days. Experiments were performed when neuronal networks were 3–6 weeks *in vitro* that permitted their functional and structural maturation (Eytan et al., 2003).

### ELECTROPHYSIOLOGY

**FIGURE 1:**
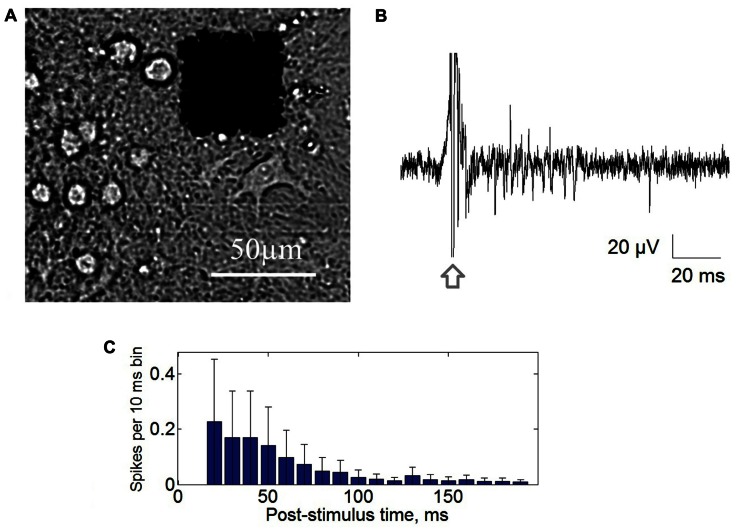
**(A)** Cultured hippocampal cells grown on multielectrode array with 64 square electrodes with 50 μm size and 150 μm inter-electrode distance. **(B)** Post-stimulus response recorded from single electrode. Stimulation artifact marked by arrow. **(C)** Post-stimulus time histogram of the recorded spikes. Each bar represents average spike rate in 10 ms post-stimulus time interval ± standard deviation.

Extracellular potentials were collected through 64 planar platinum black electrodes simultaneously with the integrated MED64 system (Alpha MED Science, Japan). The 8 × 8 (64) microelectrode arrays with 50 μm × 50 μm size and the 150 μm spacing were used for recording at sampling rate of 20 kHz/channel (**Figure 1A**). Stimuli were generated using a four channels voltage/current stimulator (STG4004, MultiChannel Systems, Germany). Closed-loop conditions were performed by custom made software (Labview^®^) using real-time signal analysis and conditional stimulation.

### SPIKE DETECTION

Detection of recorded spikes was based on threshold calculation of median of the signal according to the following formula:

(1) $$T=N_{s}\sigma ,\sigma =median\left(\frac{\left|x\right|}{0.6745}\right)$$

where *x* is the bandpass-filtered (0.3–8 KHz) data signal, σ is an estimate of the median normalized on the standard deviation of signal with zero number of spikes (Quiroga et al., 2004), and *N*_*S*_ is a spike detection coefficient determining detection threshold (Pimashkin et al., 2011). Standard deviation of signal containing Gaussian noise was equal to median of absolute values of the signal divided by 0.6745 which was a normalization of the median on the standard deviation.

Spike detection coefficient *N*_*S*_ permitted to take into account the contribution of different spike amplitudes. *N*_*S*_ = 4 was used for all data accounting spikes with amplitudes more than 20 μV. Minimal interspike interval was set to 1 ms. Detected spikes were then plotted in a raster diagram.

### STIMULATION PROTOCOL

We used trains of biphasic rectangular voltage pulses (600 mV and 300 μs per phase, with positive phase first) at low-frequency in the range of 0.05–0.06 Hz. The value of stimulation frequency was chosen to induce bursting activity in the 20–500 ms post-stimulus interval (**Figure 1B**). Note that in previous studies the stimulation frequencies were significantly higher (0.1 Hz, 0.3 Hz, Shahaf and Marom, 2001; 0.2–0.33 Hz, le Feber et al., 2010) without any relation to spontaneous bursting frequency. In our experiments most of the stimuli with frequencies higher than 0.1 Hz did not evoked stable bursting activity. However, we found that stimulation at 0.05 Hz and/or 0.06 Hz which is close to characteristic bursting frequency led to the evoked bursts. Note also, that technically the lower frequencies were also more preferable for the long-term stimulation because of the less influence on electrode disruption due to electrolysis.

Similarly, to previous studies we characterized the response by the response-to-stimulus ratio (R/S) calculated for each response and for each electrode. For our purpose, we counted the number of spikes detected in 40–80 ms post-stimulus interval on each electrode independently and then we defined R/S as the moving average across 10 preceding responses (Shahaf and Marom, 2001). This quantity indicated slow changes of the neuronal response over past 170–200 s.

#### Control stimulation (open-loop)

The control stimulation was performed during 75 min (five cycles of 10 min – stimulation, 5 min – rest) with 0.05 Hz stimulation frequency (150 stimuli) and with 0.06 Hz (180 stimuli). In more than 50% of the experiments (14 out of 24) the control stimulation was performed for 31 cycle (465 min ~7.5 h) to test the learning effect without reinforcement. After control stimulation the R/S values were calculated for each electrode.

The stimulation electrode first was chosen at random. If it evoked bursts recorded by the most of electrodes during stimulation for 5 min then the electrode was considered as stimulation electrode. If no bursting response was found, we tried another one. We considered only stably responding cultures, which during control stimulation did not significantly increase or decrease the total number of spikes in 20–300 ms post-stimulus interval for all recording electrodes. Slow changes of the responses were tested by estimating significant difference between the responses in the first and the last half of the recordings by Mann–Whitney rank-sum test (*p* < 0.05). If the sets of responses were not significantly different then the stimulation electrode was retained for further training, otherwise, we tested another electrode also chosen randomly or took another culture for the experiments. We also note, that most the cultures, in which the responses increased or decreased during control stimulation, demonstrated stable responses after several days. The responses were compared by relative changes of the mean value and of the standard deviation of the first and the last 30 stimuli responses in 20–300 ms interval normalized to the number of the spikes in the first 30 responses. Recording from each electrode was characterized by two statistical indicators: mean R/S value, M(R/S), and the R/S standard deviation, σ(R/S). The electrode for training was randomly chosen among the electrodes having M(R/S) value in the range of 0–8 with standard deviation in the range 0.1M(R/S) < σ (R/S) < 2 M(R/S) in control stimulation.

#### Training stimulation (closed-loop)

**FIGURE 2:**
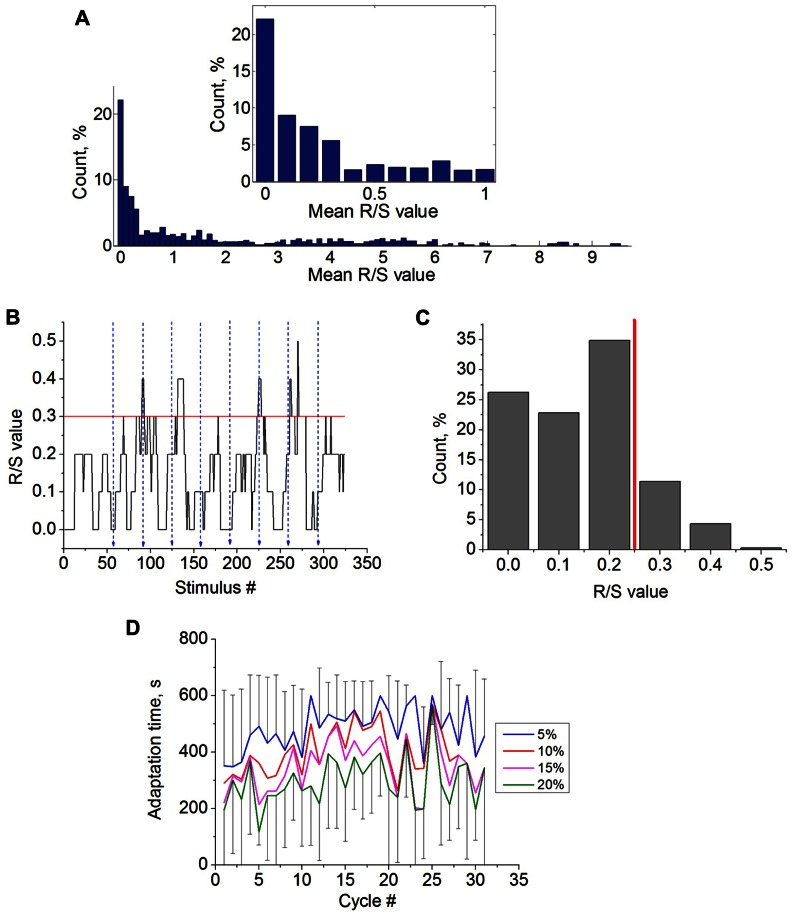
**(A)** Average R/S values (M(R/S)) distribution for all recording electrodes during low-frequency stimulation (14 trials, *n* = 10 cultures). The inset shows the magnification of the histogram in the interval [0,1] of R/S values. **(B)** R/S values during several cycles of the control stimulation at selected electrode. Blue dashed lines illustrate the ends of the stimulation cycle. Red line shows the estimated R/S threshold value (see Materials and Methods). **(C)** Distribution of the R/S values from the selected electrode during control stimulation. Red line illustrates estimated R/S threshold value (R/S_*Thr*%_ = 15%). **(D)** Learning curves calculated for control experiments using different threshold estimation parameter R/S_Thr% _- 5, 10,15, and 20% (*n* = 14; see methods). Due to high variability of the characteristics the error bars (standard deviations) illustrated only for curves with R/S_*Thr*%_ = 5% and R/S_*Thr*%_ = 20%.

Training stimulation was applied in closed-loop conditions. It started in one hour after the control stimulation. The training consisted of cyclic stimulation with continuous evaluation of the response. If the R/S value of the response to current stimulus exceeded a definite threshold then the stimulation stopped automatically. It provided the reinforcement for the culture targeting to achieve a required state. We introduced novel algorithm defining the R/S threshold for the reinforcement condition taking into account the responses in control stimulation. Such definition set different threshold values for different parts of the culture (e.g., different electrodes) involved in the training experiment. We took the highest 15% of the R/S values distribution for selected electrode, which was observed in control stimulation (example in **Figure 2C**). The lower boundary of that fraction of the distribution was assigned as the R/S threshold value. The threshold may also be referred as the 85th percentile. The percentage of the R/S values used for threshold estimation was defined as *threshold estimation parameter *R/S_*Thr*%_.

The training phase of the experiment consisted of sequence of the stimulation cycles with the same frequency and real-time evaluation of the R/S value on the selected electrode. If the R/S value of the activity from the selected electrode in response to stimulus reached the R/S threshold or if the stimulation time exceeds 10 min, then the stimulation was automatically stopped for 5 min completing the training cycle. Then the training cycle was repeated for 30–35 times. Thus the response of the neurons on the selected electrode altered the stimulation duration in each cycle. Time interval, from the beginning of the cycle to the moment where R/S value was found to be greater or equal to the R/S threshold was defined as *adaptation time*, T_R/S_. The T_R/S_ was monitored for each cycle and the sequence of T_R/S_ values defined *learning curve*. Relative change of the T_R/S_ during the experiment was defined as *adaptation time ratio*, K(T_R/S_) and was estimated as mean T_R/__S_ in the last 10 cycles divided to the mean of the T_R/__S_ in the first 10 cycles. The decrease of the T_R/S_ during the stimulation cycles [K(T_R/S_) < 0.5] was then treated as successful learning for the neurons on the selected electrode to generate the desired response on the stimulation. To compare the efficiency of the closed-loop stimulation parameters K(T_R/S_) and T_R/S_ were also calculated for control stimulation (e.g., the open-loop).

We also checked if the learning effect is stable in 4–6 h after the experiments by performing four cycle training stimulation.

At the longer time intervals (days or weeks) the cultures were changed significantly due to spontaneous development. In our experiments we reused some of them in not less than 2 days after the last training stimulation. When multiple experiments were performed on a single culture, we selected electrodes from different regions of the array for each new experiment to avoid possible influence of the previous stimulation experiments.

#### Spontaneous activity analysis

To analyze the effect of the stimulations on the state of the culture network we recorded spontaneous bursting activity during 10 min. We compared the average inter burst intervals, average number of spikes per burst and burst durations for the recordings before and after the stimulation experiments. Individual bursts detection was based on threshold estimation of basal spike rate activity as a total number of spikes observed in each 50 ms time bin (see Pimashkin et al., 2011 for more details). Statistical analysis of the bursting activity characteristics was performed by Mann–Whitney rank-sum test (*p* < 0.05).

## RESULTS

### OPEN-LOOP STIMULATION

First we analyzed responses of the culture on long-lasting (five cycles – 75 min and 31 cycles – 465 min) low-frequency stimulation (0.05, 0.06 Hz) of the stimulation electrodes that evoked population bursting response (see Materials and Methods). The stimuli were initially delivered through one randomly chosen electrode (**Figure 1B**). The dynamics of the evoked network response recorded from all electrodes was characterized by post-stimulus time histogram (PSTH). For each 10 ms time interval after the stimulus artifact a total number of the spikes recorded from all electrodes was calculated (**Figure 1C**). Maximum of the spike rate of the response was observed at 50–100 ms after stimulus.

Then, we analyzed the characteristics of the responses in the control stimulation (**Figure 2**). In our experiments we found that 31.13% of the electrodes (total 64) had 0 < M(R/S) ≤ 0.1 during the control stimulation (14 trials of long recordings, *n* = 10 cultures). The percentage of electrodes having 0 < M(R/S) ≤ 10 was 58.16% (**Figure 2A**). Particular electrodes for training stimulation were chosen among the electrodes with 0 < M(R/S) ≤ 8 (see Materials and Methods). Note that in previous studies only the activity from the electrodes with average R/S values during the control stimulation M(R/S) equal to 0.1 were chosen for training, and R/S = 0.2 was set as the R/S threshold (Shahaf and Marom, 2001; Li et al., 2007; Stegenga et al., 2009; le Feber et al., 2010).

Time dynamics of the R/S values for each stimulus response during the control stimulation is shown in **Figure 2B**. Ending moments of the 10 min stimulation cycles are marked by blue lines. Note that the responses were quite variable. The learning threshold was defined as the lower value from the highest 15% of R/S values referred as the 85th percentile of the R/S values (see Materials and Methods). The example of the R/S values distribution from the selected electrode and the R/S threshold is shown in **Figure 2C**. In other words, the threshold was set to detect quite rare and high rate responses. Note that for different electrodes the R/S thresholds were in range from 0.2 to 12 in different experiments.

After the threshold was defined the adaptation time *T*_*R/S*_ can be estimated for each cycle. To confirm that the learning effect can be induced only in closed-loop conditions, we estimated a learning curve (*T*_*R/S*_ for each cycle) for control stimulation (**Figure 2D**). The results show that adaptation time remains relatively stable. Next we analyzed the influence of the R/S threshold estimation parameter on the adaptation dynamics by setting different R/S_*Thr*%_ – 5, 10, 15, and 20% (95th, 90th, 85th, and 80th percentile, respectively). Note that the lower threshold is set the easier to reach the threshold by spontaneous fluctuations of the response. Hence, the adaptation curves for the lower thresholds were located lower (**Figure 2D**). However, changing the threshold did change qualitatively the adaptation dynamics.

### CLOSED-LOOP STIMULATION

Next we made the experiments on training stimulation with the reinforcement (see Materials and Methods). In these conditions the stimulation were turned off when the learning threshold was reached at each cycle.

**FIGURE 3:**
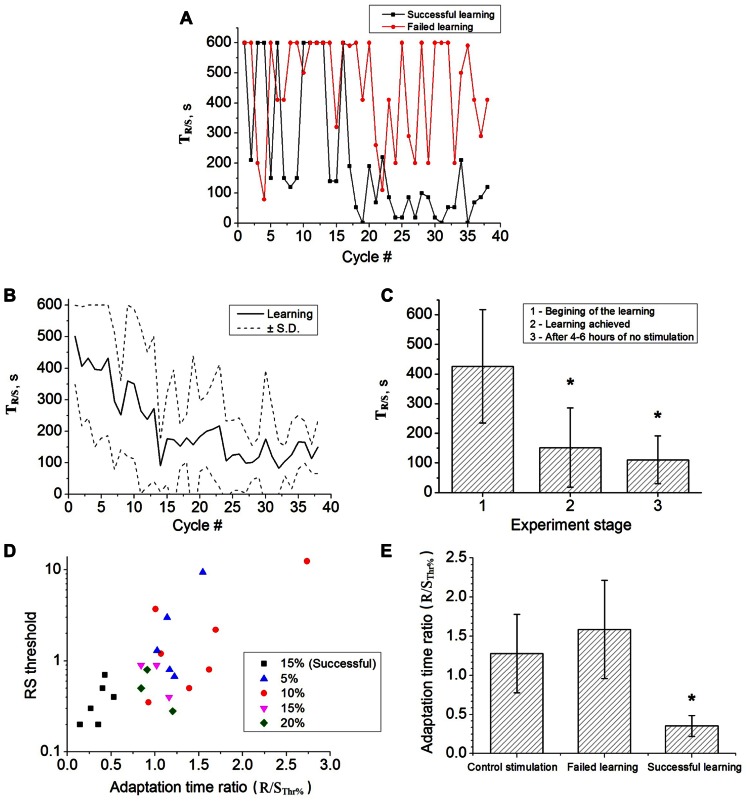
**(A)** Learning curve in one successful experiment (black curve) and failure of learning in the other experiment (red curve). In the successful case the adaptation time significantly decreased. **(B)** Average learning curve (six experiments, six cultures). Dashed curves illustrates the standard deviation **(C)** Average adaptation time during five cycles at the beginning of the training experiment (1, *n* = 6), when learning is achieved (2, *n* = 6) and in 4–6 h after the end of main experiment (3, *n* = 3). Error bar corresponds to the standard deviation, statistical significance was tested by *t*-test (*p* < 0.05). **(D)** The R/S threshold values for successful (black markers) and failed (colored markers) learning experiments. The colored markers correspond to the use of different values of the threshold estimation parameter, R/S_*Thr*%_ = 5, 10, 15, and 20% of R/S (see methods). In six experiments the learning was achieved using R/S threshold parameter 15% (out of 9 and out of 24 experiments in total). **(E)** Average adaptation time ratio for control stimulation, failed learning and successful learning. Error bar corresponds to standard deviation. The ratios of the successful learning were significantly different to the control stimulation (*t*-test, *p* < 0.05).

The adaptation dynamics for one experiment is shown in **Figure 3A**. The adaptation time for the case of successful learning (black curve) went down after several cycles of the training stimulation. We also found that some of the cultures could not be trained as illustrated by the red curve in **Figure 3A**. For those cultures the adaptation time was fluctuating with its maximal value for the whole duration of the stimulation. The training stimulation was applied for 17 different cultures in 24 experiments. **Figure 3B** shows average learning curve for the set of successful experiments. In contrast to the open-loop case (control stimulation) the adaptation time decreased indicating the learning effect. To quantify it we used the adaptation time ratio K(T_R/S_; see Materials and Methods). If K(T_R/S_) was lower than 0.6 then the training was considered as successful. We also analyzed the influence of the threshold estimation parameter (**Figure 3D**). Interestingly, that only the use of R/S_*Thr*%_ = 15%, induced the learning effect (black squares in **Figure 3D**). It was found in six of nine experiments for *n* = 9 cultures with absolute value of the R/S threshold less than 1. Similar statistics of about 50% successful experiments were reported in the previous studies (Shahaf and Marom, 2001; le Feber et al., 2010).

In the adaptation dynamics the decrease of time *T*_*R/S*_ was typically observed after 10–14 stimulation cycles (see **Figures 3A,B**). In two experiments it was decreased almost immediately after the second stimulation cycle. In average at the end of the training experiment the adaptation time became 110.62 ± 81.17. Note, that the average R/S values for the first and for the last 30 stimuli were not statistically different. In several experiments after 2–4 h of stimulation we obtained rather high *T*_*R/S*_ values leading to higher deviations in the averaged values (**Figure 3B**).

To confirm that learning effect of the closed-loop stimulation may induced long-term changes (at the time scale of hours) we performed several experiments after main course of learning. The training stimulation of four cycles (60 min) was applied in 4–6 h after end of the main experiments. We found that in three of six cases the learning effect was preserved as illustrated in **Figure 3C**.

**FIGURE 4:**
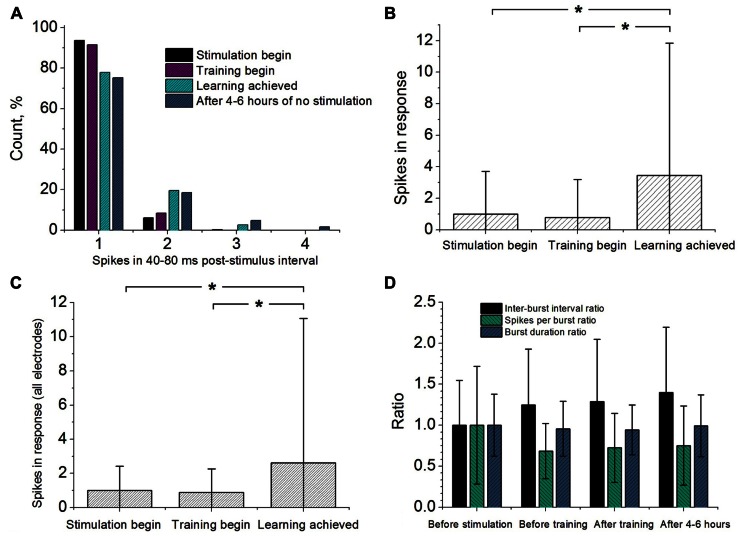
**(A)** Response statistics of the neurons from training electrode represented by the number of spikes recorded in 40–80 ms post-stimulus interval. The responses were taken from 100 stimuli in the beginning of the control stimulation, beginning and ending of the training stimulation. Number of evoked spikes in 40–80 ms post-stimulus interval recorded from selected electrode **(B)** and from all recording electrodes **(C)**. The responses were taken from 100 stimuli in the beginning of the control stimulation, beginning and ending of the training stimulation. The quantities of the spikes were normalized to ones measured in control stimulation. **(D)** Spontaneous activity changes measured before the control stimulation, before and after training stimulation and after 4–6 h after main learning experiments. The quantities of the inter-burst intervals, spikes per burst and burst durations were normalized to ones measured in control conditions (before stimulation). The values were compared with the Rank-sum test (*p * < 0.05).

Next we addressed the question if the pattern of the response is changed due to the stimulation. We analyzed changes in the number of spikes recorded in the evoked response. **Figure 4A** illustrates these changes in one of the successful experiments. One can note that after learning the spike intensity of the response increased, e.g., more responses composed of doublets, triplets and more spikes were observed. The average increase over all successful experiments is illustrated in **Figure 4B**. We also analyzed the response from other responding electrodes as illustrated in **Figure 4C**. We found that after successful learning the activity of the whole culture network increased significantly.

Changes in spontaneous activity were monitored by 10 min recordings (see Materials and Methods). We calculated the average inter burst interval, average spikes per burst and burst duration as shown in **Figure 4D**. For each characteristic we did not find any significant difference comparing between the four different phases of the experiment (before the control stimulation, before and after training stimulation and after 4–6 h after main learning experiments).

## DISCUSSION

We applied low-frequency stimulation to hippocampal culture network with on-line monitoring of the response-to-stimulus ratio (R/S) in open-loop and closed-loop conditions. The key response indicator was defined as average number of post-stimulus spikes per 10 stimuli in 40–80 ms time interval. Note that this interval corresponded to a peak in the post-stimulus histogram.

We found that learning in culture network can be achieved using an adaptive activity dependent reinforcement condition defined by the response-to-stimulus ratio (R/S) threshold value calculated from the statistics of control (e.g., the open-loop) stimulation. The threshold was estimated from the appearance of rare and high-rate responses in control stimulation (e.g., the highest 15% of the R/S values). Such responses may be associated with signal propagation along spontaneously activated and relatively strong synaptic pathways in the culture network. In other words, learning in our experiments means that particular synaptic pathways relative to particular stimulation electrode became “strengthen” to satisfy the reinforcement condition. In contrast to the previous studies in our approach we can use the electrodes with quite high basal activity in control simulation, 0 < M(R/S) < 0.5. Note, that total number of such electrodes was quite high, 67 ± 11%, which indicates that the learning protocol can be applied to rather large number of electrodes. Statistics of successful trials was about 50% which is comparable to earlier studies (Shahaf and Marom, 2001; le Feber et al., 2010).

Note, the R/S threshold, in fact, defines the reinforcement condition which is crucial for successful learning. In particular, we found that for lower values of the R/S threshold the learning effect was not achieved at all (**Figure 3D**). It is explained by the fact that the high variability of basal responses in culture network led to the increase of the fraction of random over-threshold responses that fails the learning effect which is concerned with regular changes in synaptic pathways in the network.

It is believed that learning effect is associated with structural and functional plasticity of underlying neuronal networks. In simple words synaptic connections are modified due to closed-loop stimulation to achieve an adaptive state defined by the reinforcement condition. In earlier studies low-activity electrodes were typically used (Shahaf and Marom, 2001; le Feber et al., 2010). Their activation implied that synaptic connections accompanying the electrodes were strengthened after the stimulation. Our results eventually demonstrated that not only weak connections between stimulating and recording electrodes can be increased but also well-functioning synaptic pathways can be modified for active electrodes.

Previous studies (Shahaf and Marom, 2001; Li et al., 2007) demonstrated that such training was quite selective. Only neurons on the trained electrodes increased the number of spikes in the response and hence the R/S value. In our experiments we found some increase of the responses from all electrodes (**Figure 4C**) and increase from the trained (selected) electrode (**Figure 4B**). However, the difference of this increase was not significant indicating the absence of the selectivity. We assume that it happened because of the overall activity (mean R/S) and R/S threshold were higher than in the previous studies. Setting higher reinforcement conditions for reaching the threshold in our learning protocol may require stronger modification of the overall synaptic connectivity (hence lower selectivity) to achieve learning.

Another important question was for how long time the synaptic changes can be preserved in the network after learning. We checked the response of our six trained cultures after 4–6 h and found that learning effect preserved in three of six samples (**Figures 3C,E**). Thus, the training stimulation in closed-loop conditions may induce long-term changes in structure and functions of culture network synaptic connectivity. We also found that spontaneous activity of the trained cultures was relatively stable and did not change significantly after learning experiments, e.g., we did not find statistical difference in the characteristics of the spontaneously generated bursts (inter-burst intervals, spikes per burst and burst durations).

## Conflict of Interest Statement

The authors declare that the research was conducted in the absence of any commercial or financial relationships that could be construed as a potential conflict of interest.
